# No “Wearing‐Off Effect” Seen in Quarterly or Monthly Dosing of Fremanezumab: Subanalysis of a Randomized Long‐Term Study

**DOI:** 10.1111/head.13994

**Published:** 2020-10-28

**Authors:** Andrew M. Blumenfeld, Darko M. Stevanovic, Mario Ortega, Joshua M. Cohen, Michael J. Seminerio, Ronghua Yang, Bo Jiang, Stewart J. Tepper

**Affiliations:** ^1^ The Headache Center of Southern California, The Neurology Center Carlsbad CA USA; ^2^ Teva Branded Pharmaceutical Products R&D, Inc. West Chester PA USA; ^3^ Department of Neurology Geisel School of Medicine at Dartmouth Hanover NH USA

**Keywords:** migraine, preventive, wearing‐off, calcitonin gene‐related peptide antagonist

## Abstract

**Objective:**

To evaluate whether quarterly or monthly administration of fremanezumab for migraine prevention exhibits a pattern of decreased efficacy toward the end of the dosing interval (wearing‐off effect).

**Background:**

The main goals of migraine preventive treatment are to reduce the frequency, severity, and duration of migraine attacks, and migraine‐associated disability. Wearing‐off refers to the phenomenon whereby clinical symptoms return or worsen before the next dose of a drug is due and has been reported previously with migraine preventive medications.

**Design and Methods:**

This was a long‐term, 12‐month, multicenter, randomized, double‐blind, parallel‐group phase 3 study (NCT02638103) that included chronic (CM) and episodic migraine (EM) patients who rolled over from the 12‐week phase 3 HALO CM (NCT02621931) and EM trials (NCT02629861), as well as an additional subset of 312 new patients. Patients with CM or EM received fremanezumab either monthly or quarterly. In this post hoc analysis, for selected months, the difference in the average number of migraine days between weeks 1‐2 and weeks 3‐4, between weeks 1‐3 and week 4, and between weeks 1‐2 and weeks 11‐12 were calculated.

**Results:**

A total of 1890 patients (CM, 1110; EM, 780) were enrolled. At months 3, 6, 9, and 15, there were no substantial differences in mean weekly migraine days between weeks 1‐2 and weeks 3‐4 or between weeks 1‐3 and week 4 with quarterly or monthly fremanezumab in the CM or EM subgroups. There were no substantial increases in mean weekly migraine days between weeks 1‐2 and weeks 11‐12 during the first quarter of treatment (months 1‐3) or the second quarter of treatment (months 4‐6) with quarterly or monthly fremanezumab in the CM or EM subgroups. Across both dosing subgroups in CM and EM patients, the mean weekly number of migraine days decreased substantially (30%‐42%) during the first 2 weeks; decreases in weekly migraine days remained steady during the last 2 weeks of the first quarter, with a similar maintenance of response during the second quarter.

**Conclusions:**

This analysis of data from a long‐term, phase 3 study showed that patients receiving quarterly fremanezumab or monthly fremanezumab did not experience a wearing‐off effect toward the end of the dosing interval.

**Abbreviations**
AE: adverse event
CGRP: calcitonin gene‐related peptide
CI: confidence interval
CM: chronic migraine
EM: episodic migraine
ICHD‐3 beta: *International Classification of Headache Disorders, Third Edition, beta version*
SD: standard deviation

## Introduction

Migraine is a highly disabling neurologic disease marked by recurrent headaches, which is associated with significant disability and reduced quality of life.^1^, ^2^, ^3^ The burden of impairment in daily activities and associated comorbidities increases with increased frequency of headache.^1^ Accordingly, preventive migraine treatment is recommended for patients who have 6 or more headache days per month, 4 or more headache days per month with at least some impairment, or 3 or more headache days per month with severe impairment.^3^, ^4^ Unfortunately, many people with migraine who are candidates for preventive therapy do not receive it, suggesting that preventive treatment is underutilized.^3^

Monoclonal antibodies targeting the calcitonin gene‐related peptide (CGRP) pathway are a newer class of preventive therapy that specifically target the pathophysiology of migraine.^5^, ^6^, ^7^ Advantages over oral migraine preventive medications include not requiring dose titration, long half‐lives enabling monthly or quarterly administration, and favorable safety and tolerability profiles.^4^, ^5^, ^8^ As a class, these monoclonal antibodies targeting the CGRP pathway have been shown to be effective in reducing the frequency of migraine days.^9^ Fremanezumab, a fully humanized monoclonal antibody (IgG2Δa)^10^ that selectively binds to the CGRP ligand, is approved in the United States, the European Union, and several other countries for the preventive treatment of migraine in adults.^11^, ^12^, ^13^, ^14^, ^15^ Fremanezumab is the only monoclonal antibody targeting the CGRP pathway approved for both monthly and quarterly subcutaneous dosing. The efficacy and safety of fremanezumab were demonstrated in phase 2 studies^16^, ^17^ and in 2 pivotal 12‐week, randomized, double‐blind, placebo‐controlled phase 3 efficacy studies in patients with chronic migraine (CM; 15 or more headache days per month, at least 8 of which were migraine days; HALO CM)^18^ and episodic migraine (EM; fewer than 15 headache days per month; HALO EM).^19^ Fremanezumab also demonstrated efficacy in patients with difficult‐to‐treat migraine who had experienced inadequate response to up to 4 different classes of migraine preventive medications.^20^ Patients from the initial HALO CM and EM trials had the option of continuing treatment in a 12‐month, phase 3 study (“rolling over”), and additional patients were directly enrolled in this long‐term study. Results from that study confirmed that fremanezumab is generally well tolerated and provides sustained improvements in monthly migraine days, headache days, and headache‐related disability for up to 15 months of treatment (including both the 3‐month, parent HALO study and the 12‐month study).^21^ Across these phase 3 trials, fremanezumab demonstrated similar treatment effects with both quarterly and monthly dosing regimens.^18^, ^19^, ^20^, ^21^

A “wearing‐off” effect, described as the return or worsening of clinical symptoms before the next dose of a drug and improvement after the next dose, has been reported in patients taking some types of preventive migraine treatment with long dosing intervals.^22^ Given the relatively long intervals between doses with both dosing regimens of fremanezumab, it is important to understand whether there is any evidence of wearing‐off between doses. The objective of this analysis was to evaluate whether wearing‐off, defined as reduced efficacy of a drug in the final weeks before the next scheduled dose, was observed with the quarterly or monthly dosing regimens of fremanezumab during up to 15 months of treatment. Based on the sustained clinical benefit with fremanezumab observed in the long‐term safety study, we hypothesized that quarterly and monthly dosing of fremanezumab would not demonstrate a wearing‐off effect prior to the next scheduled dose.

## Methods

### Study Design

The design of the long‐term study has been described previously.^21^ This was a 12‐month, multicenter, randomized, double‐blind, parallel‐group phase 3 study (Clinicaltrials.gov Identifier: NCT02638103) that included 917 and 661 patients who rolled over from the 12‐week phase 3 HALO CM (NCT02621931) and EM trials (NCT02629861), respectively. An additional subset of 312 new patients who were not previously enrolled in the HALO trials were directly recruited into the long‐term study. Therefore, up to 15 months of data were available for patients who rolled over from the HALO CM and EM trials, while up to 12 months of data were available for the 312 new patients who enrolled in the long‐term study. The study consisted of a screening visit, a 28‐day run‐in period (for new patients only), a 12‐month double‐blind treatment period, and a 6.5‐month follow‐up period for antidrug antibody assessment. Based on screening and pretreatment daily diary information prior to the HALO CM and EM trials, patients were randomized into the appropriate trial or were excluded.

The long‐term study was conducted in accordance with the International Conference on Harmonisation guidelines for Good Clinical Practice, principles of the Declaration of Helsinki, and local and national regulations. The protocol was approved by the relevant national/local health authorities and each Independent Ethics Committee/Institutional Review Board. All patients provided written informed consent.

### Patients

Eligible patients were adults aged from 18‐70 years, with a history of migraine (according to *International Classification of Headache Disorders Third Edition, beta version* criteria [ICHD‐3 beta])^23^ for at least 12 months before screening. Patients were prospectively classified as having CM or EM based on headache data recorded daily in an electronic headache diary device during the 28‐day run‐in period. CM was defined as headache occurring on at least 15 days, with at least 8 days fulfilling ICHD‐3 beta criteria for migraine, probable migraine, or use of triptan or ergot medications. EM was defined as headache occurring on 6‐14 days (rollover patients) or 4‐14 days (new patients), with at least 4 days fulfilling ICHD‐3 beta criteria for migraine, probable migraine, or use of triptan or ergot medications. Patients could continue using a maximum of 1 (rollover patients) or 2 (new patients) concomitant migraine preventive medications at a stable dose for the duration of the study, provided that the medication was recognized as having at least moderate efficacy in the preventive treatment of migraine and dosing had been stable for at least 2 consecutive months before screening. Patients rolling over from the previous HALO CM or EM trials were excluded if they had used onabotulinumtoxinA in the 4 months before screening, opioids or barbiturates on more than 4 days per month during the pretreatment period, or interventions or devices for migraine in the 2 months before screening. Patients were also excluded if they experienced previous failure in at least 2 of the following medication clusters after at least 3 months of treatment: divalproex sodium and sodium valproate; flunarizine and pizotifen; amitriptyline, nortriptyline, venlafaxine, and duloxetine; or atenolol, nadolol, metoprolol, propranolol, and timolol. These exclusion criteria did not apply to new patients.

**Fig. 1:**
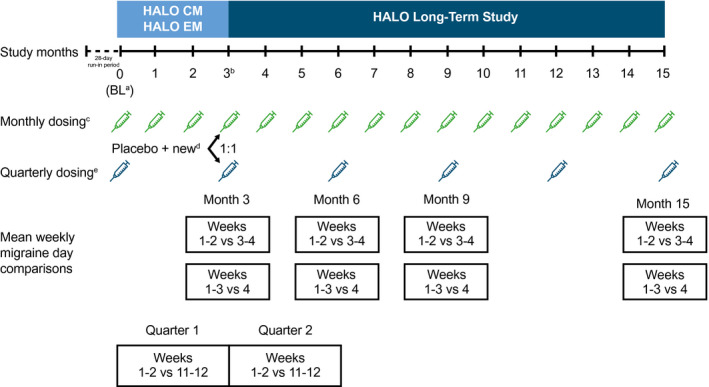
Study design and wearing‐off analysis assessment time points. Chronic migraine (CM); episodic migraine (EM); baseline (BL). ^a^For the patients included in these analyses (rollover patients initially randomized to fremanezumab in the HALO CM or EM studies), baseline is the 28‐day run‐in period (for headache variables) or Day 0 for the HALO CM or EM studies. ^b^End‐of‐treatment assessments for the HALO CM or EM studies were completed prior to starting any procedures/assessments for the HALO long‐term study. ^c^Monthly doses were administered approximately every 28 days. For the first dose of monthly treatment, patients with CM received 675 mg, and patients with EM received 225 mg. All subsequent monthly doses were 225 mg. ^d^Rollover patients who received placebo during the initial HALO CM and EM trials, as well as new patients, who were randomized 1:1 to quarterly or monthly fremanezumab at the start of the long‐term study were excluded from these wearing‐off analyses. ^e^For quarterly dosing, patients received 675 mg at the start of each quarter, then placebo during the remaining 2 months of each quarter.

Additional details regarding study design and dosing intervals are illustrated in Figure 1.

### Study Treatment

In the initial placebo‐controlled HALO CM and EM trials, patients were randomized 1:1:1 to receive subcutaneous injections of 1 of the following treatments approximately every 28 days (28 ± 5 days), for a total of 3 doses: quarterly fremanezumab (675 mg at baseline and placebo at weeks 4 and 8), monthly fremanezumab (CM: 675 mg at baseline and 225 mg at weeks 4 and 8; EM: 225 mg at baseline and at weeks 4 and 8), or placebo at baseline and at weeks 4 and 8. In the long‐term trial, patients who received active treatment in the prior placebo‐controlled trial continued the same treatment, while patients who previously received placebo and new patients were randomized 1:1 to quarterly or monthly fremanezumab. All patients remained blinded as to which dosing regimen they received during the long‐term study.

### Outcomes

Efficacy endpoints for this post hoc analysis were the mean weekly number of migraine days during weeks 1‐2 and weeks 3‐4 at months 3, 6, 9, and 15; during weeks 1‐3 and week 4 at months 3, 6, 9, and 15; and during weeks 1‐2 and weeks 11‐12 of the first and second quarters (months 1‐3 and months 4‐6) of treatment (Fig. 1). A migraine day was defined as a calendar day with either at least 2 (EM) or 4 (CM) consecutive hours of a headache meeting criteria for migraine (with or without aura); probable migraine (only 1 migraine criterion absent); or a day, regardless of duration, when acute migraine‐specific medication was used to treat a headache.

In the long‐term study, protocol defined efficacy analyses were only performed at months 1, 2, 3, 6, and 12. Consequently, analyses points for end‐of‐quarter dosing were only available at months 3, 6, 9, and 15. Changes in the mean weekly number of migraine days from weeks 1‐2 to weeks 3‐4 and from weeks 1‐3 to week 4 at months 3, 6, 9, and 15, as well as changes in the mean weekly number of migraine days from weeks 1‐2 and weeks 11‐12 for the first and second quarters of treatment, were also evaluated.

Safety and tolerability endpoints included adverse events and systematic local injection‐site assessments (immediately and at 1 hour post‐injection).

### Statistical Analysis

Efficacy analyses were conducted in the full analysis set, which included all randomized patients with at least 1 post‐baseline efficacy assessment. The safety population included all randomized patients who received at least 1 dose of study drug during the study. Efficacy and safety outcomes were summarized using descriptive statistics (ie, sample size, mean, standard deviation, and frequency counts). The normality assumption was checked using visual inspections of Q‐Q plots and histograms, as well as the Shapiro‐Wilk test for all efficacy endpoints using normal approximation theories in the HALO studies. Where the validity of the assumption was suspected, nonparametric method was used as a sensitivity analysis. As expected from the large‐sample normal approximation theory, the results from the sensitivity analyses and the primary analyses were consistent, demonstrating the robustness of study results using *t* tests. Therefore, in this study, we only conducted analyses and reported results based on the normality assumption.

Efficacy outcomes for the first 3 months are presented for patients who were randomized to fremanezumab during the HALO trials. Efficacy outcomes for the remaining time points during the long‐term study are presented for patients who completed the 12‐week treatment period in the HALO trials, then rolled over to the long‐term study (total of 15 months of study treatment). Patients who newly initiated fremanezumab in the long‐term study (ie, patients who received placebo in the HALO studies or were new patients in the long‐term study) were not included in these analyses.

Wearing‐off was defined by a clinically meaningful loss of effect at the end of the dosing interval, established separately for EM and CM based upon the treatment effect seen over placebo during the double‐blind phases of HALO EM and HALO CM, respectively. Using the effect sizes over placebo of −1.3/−1.5 monthly migraine days for HALO EM and −1.7/−1.9 monthly migraine days for HALO CM, mean weekly effect sizes were established as −0.4 weekly migraine days for EM and −0.5 weekly migraine days for CM. Wearing‐off was then defined as a 50% reduction in standard effect size at the end of a dosing interval, corresponding to an increase in weekly migraine days of 0.2 for EM or 0.25 for CM.

For the changes in the mean weekly number of migraine days during the specified intervals, the mean and 95% confidence interval (CI) are presented. The 95% CI was based on a paired *t* test for the difference between the specified initial and ending weeks of each interval. All summaries and statistical analyses were generated using SAS^®^ software (Version 9.4 of SAS System for Windows, SAS Institute Inc., Cary, NC, USA).

## Results

### Study Population

**Fig. 2:**
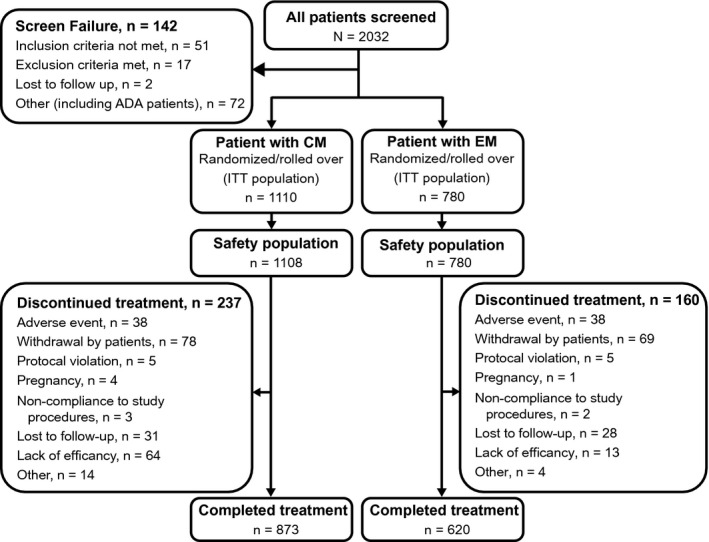
Patient disposition. Anti‐drug antibody (ADA); chronic migraine (CM); episodic migraine (EM); intent‐to‐treat (ITT).

A total of 1890 patients (1110 with CM and 780 with EM) were enrolled in the long‐term study (Fig. 2). Of the 1890 patients enrolled, 1578 had rolled over from the HALO studies (917 from the HALO CM study and 661 from the HALO EM study) and 312 were new patients (193 of whom had CM and 119 of whom had EM). Of the patients who rolled over from the HALO studies, 611 from the HALO CM study (quarterly, n = 306; monthly, n = 305) and 432 from the HALO EM study (quarterly, n = 217; monthly, n = 215) had received fremanezumab during the respective HALO study and were included in analyses of wearing‐off during the long‐term study.

Within each migraine diagnosis group in the long‐term study,^21^ baseline demographics and clinical characteristics of patients were similar across the quarterly and monthly treatment groups. Among patients receiving quarterly and monthly dosing in the long‐term study, respectively, the mean (standard deviation [SD]) age was 43.7 (12.0) and 42.6 (11.8) years among patients with CM and 43.3 (11.3) and 44.7 (12.2) among patients with EM. The majority of patients across dosing and migraine diagnosis groups were women (≥84%) and approximately a quarter of patients were currently using migraine preventive medications. The mean (SD) monthly average number of migraine days was 16.4 (5.1) days in the quarterly dosing group and 16.4 (5.3) days in the monthly dosing group for patients with CM and 9.2 (2.6) and 9.1 (2.7) days, respectively, for patients with EM.

### Assessment of Potential Wearing‐Off Effect Over the First and Second Halves of 1‐Month Intervals

**Fig. 3:**
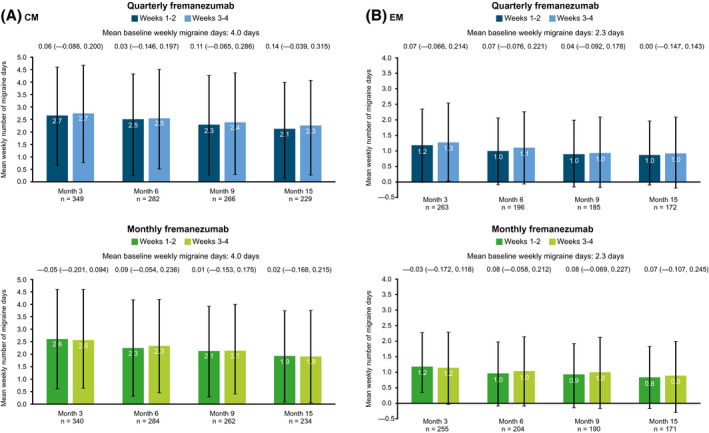
Mean number of migraine days in patients with (A) chronic migraine (CM) or (B) episodic migraine (EM), during weeks 1‐2 and 3‐4 at 3, 6, 9, and 15 months after the first injection of fremanezumab (full analysis set).^a,b^ ^a^Values above the bars are mean (95% confidence interval [CI]) difference between weeks 1‐2 and weeks 3‐4. ^b^n values shown are the patients with data available at both time points, which were used in the analyses of mean differences between weeks 1‐2 and weeks 3‐4.

Weekly migraine days at weeks 1‐2 and weeks 3‐4, along with the difference in weekly migraine days between weeks 1‐2 and weeks 3‐4, for months 3, 6, 9, and 15 are shown in Figure 3. For patients with CM taking quarterly and monthly fremanezumab, the mean (SD) weekly numbers of migraine days at baseline were 4.0 (1.2) and 4.0 (1.3), respectively, and decreased by approximately 34% to 2.7 (2.0) and 35% to 2.6 (2.0), respectively, during the first 2 weeks of treatment (Fig. 3A). For patients with EM, the mean (SD) weekly numbers of migraine days at baseline in the quarterly and monthly fremanezumab groups were 2.3 (0.6) and 2.3 (0.7) days, respectively, and decreased by approximately 48% to 1.2 (1.2) and 50% to 1.2 (1.1), respectively, during the first 2 weeks (Fig. 3B). These reductions were generally maintained through the remaining evaluated intervals. There were no substantial differences in mean weekly migraine days between weeks 1‐2 and weeks 3‐4 with quarterly or monthly fremanezumab in the CM or EM groups.

For patients with EM taking quarterly fremanezumab, mean (95% CI) differences in weekly average migraine days between the first and second halves of the month were 0.07 (−0.066, 0.214) at month 3, 0.07 (−0.076, 0.221) at month 6, 0.04 (−0.092, 0.178) at month 9, and 0.00 (−0.147, 0.143) at month 15. For those taking monthly fremanezumab, mean (95% CI) differences in weekly average migraine days between the first and second halves of the month were −0.03 (−0.172, 0.118) at month 3, 0.08 (−0.058, 0.212) at month 6, 0.08 (−0.069, 0.227) at month 9, and 0.07 (−0.107, 0.245) at month 15.

### Assessment of Potential Wearing‐Off Effect Over the First 3 Weeks and Last Week of 1‐Month Intervals

**Fig. 4:**
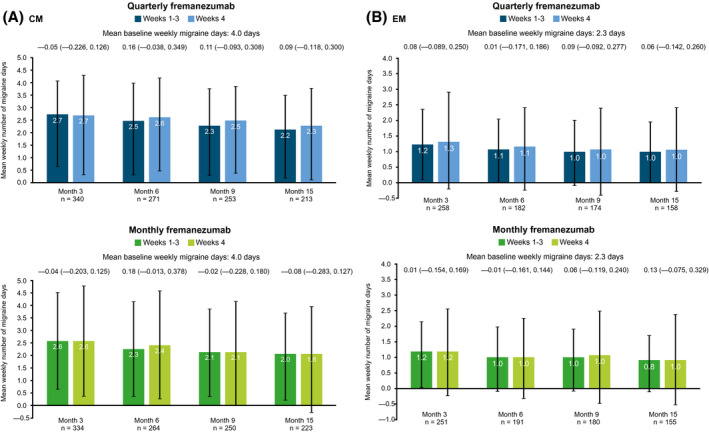
Mean number of migraine days in patients with (A) chronic migraine (CM) or (B) episodic migraine (EM), during weeks 1‐3 and week 4 at 3, 6, 9, and 15 months after the first injection of fremanezumab (full analysis set).^a,b^ ^a^Values above the bars are mean (95% confidence interval [CI]) difference between weeks 1‐3 and week 4. ^b^n values shown are the patients with data available at both time points, which were used in the analyses of mean differences between weeks 1‐3 and week 4.

Weekly migraine days at weeks 1‐3 and week 4, along with the difference in weekly migraine days between weeks 1‐3 and week 4, for months 3, 6, 9, and 15 are shown in Figure 4. For patients with CM taking quarterly and monthly fremanezumab, the mean (SD) weekly numbers of migraine days at baseline were 4.0 (1.2) and 4.0 (1.3), respectively, and decreased by approximately 32% to 2.7 (2.0) and 36% to 2.6 (1.9), respectively, during the first 3 weeks of treatment (Fig. 4A). In patients with EM, the mean (SD) weekly numbers of migraine days at baseline in the quarterly and monthly fremanezumab groups were 2.3 (0.6) and 2.3 (0.7) days, respectively, and decreased by approximately 47% to 1.2 (1.1) and 50% to 1.2 (1.0), respectively, during the first 3 weeks of treatment (Fig. 4B). These changes were generally maintained through the remaining evaluated intervals. There were no substantial differences in mean weekly migraine days between weeks 1‐3 and week 4 with quarterly or monthly fremanezumab in the CM or EM groups.

### Assessment of Potential Wearing‐Off Effect Over the First 2 Weeks and Last 2 Weeks of Quarters

**Fig. 5:**
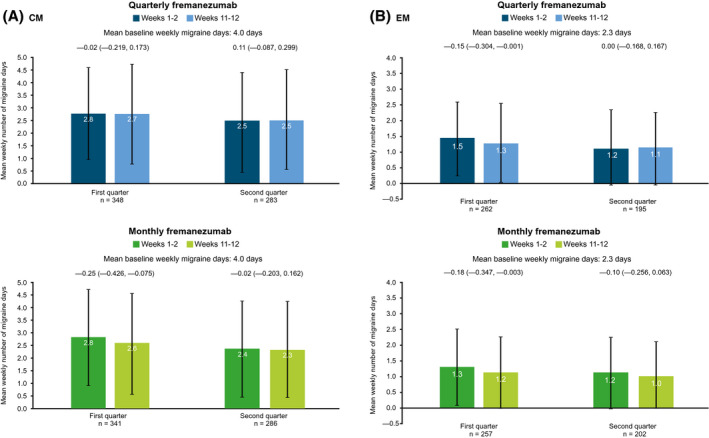
Mean number of migraine days in patients with (A) chronic migraine (CM) or (B) episodic migraine (EM), during weeks 1‐2 and weeks 11‐12 of the first quarter (months 1‐3) and second quarter (months 4‐6) after the first injection of fremanezumab (full analysis set).^a,b^ ^a^Values above the bars are mean (95% confidence interval [CI]) difference between weeks the first 2 weeks and the last 2 weeks of the quarter. ^b^n values shown are the patients with data available at both time points, which were used in the analyses of mean differences between the first 2 weeks and the last 2 weeks of the quarter.

Weekly migraine days at weeks 1‐2 and weeks 11‐12, along with the difference in weekly migraine days between weeks 1‐2 and weeks 11‐12, for the first quarter (months 1‐3) and second quarter (months 4‐6) of study treatment are shown in Figure 5. As noted previously, during the first 2 weeks of fremanezumab quarterly and monthly treatment, respectively, approximate 31% and 30% decreases from baseline in weekly migraine days were observed for patients with CM and approximately 37% and 42% decreases were observed in patients with EM. These reductions in weekly migraine days were generally maintained or increased through the remaining evaluated intervals. There were no substantial increases in mean weekly migraine days between weeks 1‐2 and weeks 11‐12 with quarterly or monthly fremanezumab in the CM or EM groups (Table 1).

**Table 1:** Weekly Number of Migraine Days

Treatment Subgroups	Chronic Migraine (n = 1110)								Episodic Migraine (n = 780)							
	Quarterly Fremanezumab†				Monthly Fremanezumab†				Quarterly Fremanezumab‡				Monthly Fremanezumab‡			
Time point	First Quarter		Second Quarter		First Quarter		Second Quarter		First Quarter		Second Quarter		First Quarter		Second Quarter	
	Wks 1‐2	Wks 11‐12	Wks 1‐2	Wks 11‐12	Wks 1‐2	Wks 11‐12	Wks 1‐2	Wks 11‐12	Wks 1‐2	Wks 11‐12	Wks 1‐2	Wks 11‐12	Wks 1‐2	Wks 11‐12	Wks 1‐2	Wks 11‐12
Weekly number of migraine days, mean (SD)	2.8 (1.9)	2.7 (2.0)	2.5 (2.0)	2.5 (2.0)	2.8 (2.0)	2.6 (2.0)	2.4 (2.0)	2.3 (1.9)	1.5 (1.2)	1.3 (1.3)	1.2 (1.3)	1.1 (1.2)	1.3 (1.3)	1.2 (1.2)	1.2 (1.2)	1.0 (1.2)
% decrease in weekly migraine days from baseline	31	32	38	37	30	36	40	42	37	44	49	51	42	50	50	56

† CM patients: mean (SD) weekly numbers of migraine days at baseline in the quarterly and monthly fremanezumab groups were 4.0 (1.2) and 4.0 (1.3), respectively.

‡ EM patients: mean (SD) weekly numbers of migraine days at baseline in the quarterly and monthly fremanezumab groups were 2.3 (0.6) and 2.3 (0.7) days, respectively.

SD = standard deviation; Wks = weeks.

### Safety and Tolerability

**Fig. 6:**
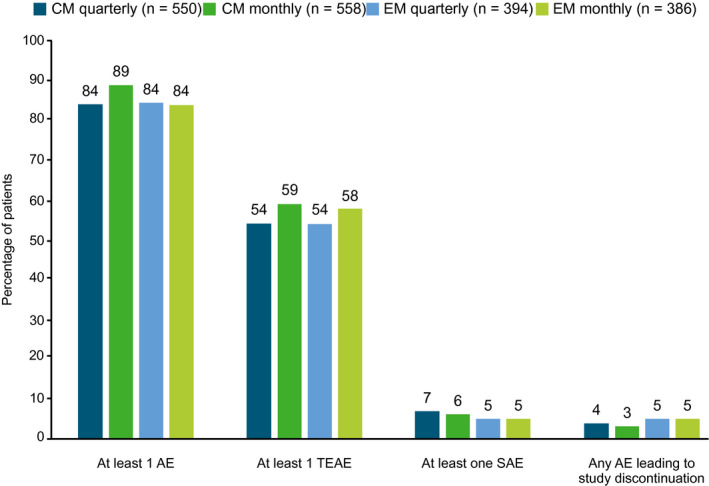
Summary of adverse events (safety population). Adverse event (AE); chronic migraine (CM), episodic migraine (EM), serious adverse event (SAE), treatment‐emergent adverse event (TEAE).

Safety and tolerability results from the long‐term study have been reported in full previously.^21^ In brief, similar proportions of patients with CM and EM in each fremanezumab treatment arm reported at least 1 adverse event (Fig. 6). The most commonly reported adverse events were injection‐site reactions, with similar incidence rates between treatment groups. The most common types of injection‐site reactions reported were injection‐site induration (CM: quarterly, 30%; monthly, 35%; EM, quarterly, 29%; monthly 38%), injection‐site pain (29%, 33%, 30%, and 32%, respectively), and injection‐site erythema (25%, 31%, 22%, and 27%, respectively). Serious adverse events and adverse events leading to discontinuation were infrequent, with similar incidences across treatment groups.

## Discussion

This analysis of migraine days over the course of the 3‐month HALO studies and 12‐month, long‐term study of fremanezumab showed no evidence of a wearing‐off effect toward the end of the dosing interval with either monthly or quarterly dosing regimens in patients with CM or EM. If fremanezumab treatment was subject to a wearing‐off effect, we might expect to see an increase in the average number of migraine days at the end of the dosing interval compared to the start of the dosing interval. However, this analysis showed no substantial increase in mean weekly migraine days over weeks 1‐2 compared with weeks 3‐4 or over weeks 1‐3 compared with week 4 during multiple 1‐month intervals (months 3, 6, 9, and 15), or at weeks 1‐2 compared with weeks 11‐12 during 2 separate 3‐month intervals (first and second quarters). Thus, these analyses showed no evidence of a wearing‐off effect at the end of the monthly or quarterly dosing intervals.

OnabotulinumtoxinA is an injected preventive treatment that has shown efficacy in reducing the frequency of migraine days in individuals with CM.^24^ However, at its approved intramuscular dosing regimen of 12‐week intervals,^25^ there is evidence that some patients experience a wearing‐off effect. Across several studies, 23%‐63% of patients reported a wearing‐off of efficacy as early as 4 weeks prior to next treatment.^26^, ^27^, ^28^ Therefore, wearing‐off has become a concern for patients and practitioners for the quarterly dosing of monoclonal antibodies targeting the CGRP pathway. It has been suggested that 12 weeks represents a mean duration of response to onabotulinumtoxinA, with some patients expected to have a shorter duration of response.^22^ Shortening the dose interval is not an approved treatment strategy for onabotulinumtoxinA, and dosage increase is currently the only option for patients experiencing wearing‐off effects.^22^

The 2 approved dosing regimens for fremanezumab are subcutaneous injection of 225 mg once monthly or 675 mg once quarterly,^11^ with the regimens demonstrating similar efficacy and tolerability profiles in patients with CM or EM.^18^, ^19^ In the 12‐week pivotal studies, approximately 40%‐50% of patients experienced a 50% or greater reduction in frequency of migraine days with quarterly fremanezumab or monthly dosing, with this effectiveness sustained over a subsequent 12 months of treatment.^21^ In addition, fremanezumab treatment was associated with significant improvements in migraine‐ and headache‐related disability.^18^, ^19^, ^21^ Most individuals with migraine rate effectiveness as the most important aspect of preventive therapy, and would prefer a treatment option with high efficacy even if it was dosed more frequently.^29^ A recent survey of 417 US adults with migraine showed that a similar proportion of patients expressed a preference for preventive treatment with a monthly or quarterly regimen (35% and 40%, respectively).^30^ The most common reasons for preferring monthly dosing included “consistent protection against migraine” and “facilitates establishment of a treatment routine,” while reasons for preferring quarterly dosing included “more convenient” and “fewer treatments to keep track of.” Patients reported that they would be more likely to adhere with the treatment regimen for which they expressed a preference. These responses suggest that migraine patients would benefit from having a choice of dosing regimens, allowing them to choose the regimen that matches their individual preferences and lifestyles.^30^

This analysis was limited to assessments only of the changes in migraine days during the dosing intervals; changes in other efficacy outcomes of the HALO studies and long‐term study were not assessed. Reductions in migraine days, however, are generally considered one of the key efficacy assessments for a migraine preventive treatment and are a critical indicator of the severity of disease.^23^ An additional limitation of the current analysis was the lack of a placebo control during the 12‐month study, but given that these analyses compared a cohort of patients to themselves at different time points on active treatment, the impact of lack of placebo on these outcomes is likely minimal.^21^ Also, all analyses were performed as descriptive statistics showing small differences between various time points. An assumption of small differences such as half of the wearing‐off thresholds, that is, 0.125 day for CM and 0.1 day for EM, the sample sizes (ranging from 155 to 348 in the comparisons) would provide limited powers (<50%) to demonstrate neither non‐inferiority (using non‐inferiority margins of 0.25 for CM and 0.2 for EM) for maintenance of treatment effects nor wearing‐off over time. Selection bias may be present, since patients lost to observation may have experienced reduced efficacy. Considering this, the post hoc study population may consist of patients who were already responsive to treatment. Lack of explanation for these lost observations or any missing data may limit full interpretation of these results. However, lack of efficacy (4%) was a rare cause of discontinuation in the long‐term study, so the impact of discontinuation on the interpretation of these results may be limited.

The goals of migraine preventive treatment include reducing attack frequency, severity, duration, and disability, and also enabling patients to manage their own disease, enhancing a sense of personal control.^4^ With demonstrated efficacy in reducing migraine frequency and related disability and a choice of dosing regimens, fremanezumab represents an important preventive option for individuals with CM or EM.

## Conclusion

This analysis of data from a long‐term phase 3 study demonstrates that patients receiving quarterly fremanezumab or monthly fremanezumab did not experience a wearing‐off effect toward the end of the dosing interval. Along with previous data showing comparable efficacy for quarterly and monthly fremanezumab,^18^, ^19^, ^21^ these analyses provide further support for the provision of these 2 dosing options to patients to meet individual needs and preferences.

## Statement of Authorship

**Category 1**

**(a) Conception and Design**

Darko M. Stevanovic, Mario Ortega, Joshua M. Cohen, Michael J. Seminerio, Ronghua Yang, Bo Jiang

**(b) Acquisition of Data**

Darko M. Stevanovic, Mario Ortega, Joshua M. Cohen, Michael J. Seminerio, Ronghua Yang, Bo Jiang

**(c) Analysis and Interpretation of Data**

Andrew M. Blumenfeld, Darko M. Stevanovic, Mario Ortega, Joshua M. Cohen, Michael J. Seminerio, Ronghua Yang, Bo Jiang, Stewart J. Tepper

**Category 2**

**(a) Drafting the Manuscript**

Andrew M. Blumenfeld, Darko M. Stevanovic, Mario Ortega, Joshua M. Cohen, Michael J. Seminerio, Ronghua Yang, Bo Jiang, Stewart J. Tepper

**(b) Revising It for Intellectual Content**

Andrew M. Blumenfeld, Darko M. Stevanovic, Mario Ortega, Joshua M. Cohen, Michael J. Seminerio, Ronghua Yang, Bo Jiang, Stewart J. Tepper

**Category 3**

**(a) Final Approval of the Completed Manuscript**

Andrew M. Blumenfeld, Darko M. Stevanovic, Mario Ortega, Joshua M. Cohen, Michael J. Seminerio, Ronghua Yang, Bo Jiang, Stewart J. Tepper
